# Mitochondrial-derived damage-associated molecular patterns amplify neuroinflammation in neurodegenerative diseases

**DOI:** 10.1038/s41401-022-00879-6

**Published:** 2022-03-01

**Authors:** Miao-miao Lin, Na Liu, Zheng-hong Qin, Yan Wang

**Affiliations:** Department of Pharmacology and Laboratory of Aging and Nervous Diseases and Jiangsu Key Laboratory of Neuropsychiatric Diseases, College of Pharmaceutical Sciences, Soochow University, Suzhou, 215123 China

**Keywords:** neuroinflammation, mitochondrial dysfunction, neurodegenerative diseases, mitochondrial-derived damage-associated molecular pattern, microglia

## Abstract

Both mitochondrial dysfunction and neuroinflammation are implicated in neurodegeneration and neurodegenerative diseases. Accumulating evidence shows multiple links between mitochondrial dysfunction and neuroinflammation. Mitochondrial-derived damage-associated molecular patterns (DAMPs) are recognized by immune receptors of microglia and aggravate neuroinflammation. On the other hand, inflammatory factors released by activated glial cells trigger an intracellular cascade, which regulates mitochondrial metabolism and function. The crosstalk between mitochondrial dysfunction and neuroinflammatory activation is a complex and dynamic process. There is strong evidence that mitochondrial dysfunction precedes neuroinflammation during the progression of diseases. Thus, an in-depth understanding of the specific molecular mechanisms associated with mitochondrial dysfunction and the progression of neuroinflammation in neurodegenerative diseases may contribute to the identification of new targets for the treatment of diseases. In this review, we describe in detail the DAMPs that induce or aggravate neuroinflammation in neurodegenerative diseases including mtDNA, mitochondrial unfolded protein response (mtUPR), mitochondrial reactive oxygen species (mtROS), adenosine triphosphate (ATP), transcription factor A mitochondria (TFAM), cardiolipin, cytochrome *c*, mitochondrial Ca^2+^ and iron.

## Introduction

Neurodegenerative diseases, which are characterized by a progressive loss of neurons, include Alzheimer’s disease (AD), Parkinson’s disease (PD) and Huntington’s disease [1]. Mitochondrial dysfunction, neuroinflammation, excitotoxicity and oxidative stress are closely associated with the pathogenesis of neurodegenerative diseases [2–4]. Although both mitochondrial dysfunction and neuroinflammation can cause neuronal death, the underlying mechanisms involved in neuronal degeneration have not been elucidated. There is growing recognition that mitochondrial dysfunction and neuroinflammation do not act alone and may be intricately linked. On the one hand, mitochondrial dysfunction is involved in the activation of neuroinflammation. However, inflammatory factors can also interfere with normal mitochondrial function. Despite some controversy, there is strong evidence indicating that mitochondrial dysfunction precedes neuroinflammation during the progression of diseases. Thus, an in-depth understanding of the specifically related mechanisms associated with mitochondrial dysfunction and the progression of neuroinflammation in neurodegenerative diseases may contribute to the identification of new targets for the treatment of neurodegenerative diseases.

Mitochondria are intracellular organelles derived from bacterial symbionts. Because of their endosymbiotic origin, mitochondria possess bacterial characteristics, such as cytosine-phosphate-guanosine, the membrane lipid cardiolipin, N-formylated peptides and circular double-stranded DNA [5]. Considering their essential roles in many physiological and pathological processes, maintaining mitochondrial health is indispensable for cell survival and function [6]. The accumulation of mitochondrial DNA (mtDNA) mutations, increased reactive oxygen species (ROS) production, changes in mitochondrial dynamics and loss of mitochondrial membrane potential exacerbate mitochondrial dysfunction. Neurons have high energy requirements and are particularly vulnerable to mitochondrial dysfunction [7, 8]. Several studies have targeted mitochondrial dysfunction to screen for effective drugs considering the role of mitochondrial dysfunction in neurodegenerative diseases [9]. In 1911, scientists discovered that the destruction of mitochondrial health and neuron metabolism was an early event in AD. AD mice exhibited decreased mitochondrial respiration at the age of 3 months. In contrast, a significant increase in β-amyloid at the age of 9 months indicated that mitochondrial dysfunction occurred at an early stage in the pathogenesis of AD [10]. Interestingly, some PD-related genes, such as *Parkin* and *PINK1*, are related to the mitochondrial quality control pathway [11]. Mitochondrial dysfunction destroys the integrity of the mitochondrial membrane and results in the release of mitochondrial ligands into the cytoplasm or out of the cell [12]. The ligands released by mitochondria are also called mitochondrial-derived damage-associated molecular patterns (DAMPs) [13]. DAMPs are endogenous molecules, but they are typically isolated by host cells and considered danger signals. They activate pattern recognition receptors and trigger the intracellular signal transduction cascade, resulting in the expression of inflammatory mediators and coordinating the elimination of pathogens and infected cells [14].

Inflammation is an immune response that protects and defends the host [15]. Neuroinflammation is the inflammatory response within the central nervous system (CNS), which is caused by infection, trauma, toxin accumulation and other pathological injuries [16]. Although neuroinflammation is a protective mechanism, excessive or prolonged neuroinflammation can result in tissue damage and disease [17, 18]. Microglia are resident macrophages in the CNS that serve as primary mediators of neuroinflammation. During injury, microglia are responsible for the phagocytosis and elimination of microorganisms, dead cells, protein aggregates, and other particles and soluble antigens that may harm the CNS [19]. They secrete cytokines and neurotrophic factors that contribute to the immune response and tissue repair of the CNS. Microglia respond to pathological events through inflammatory processes by sensing putative pathogen-associated molecular patterns or DAMPs through the expression of various immune receptors, such as pattern recognition receptors and chemokine receptors. However, the overactivation of glial cells can damage surrounding healthy neurons and factors secreted by dead or dying neurons. This subsequently exacerbates the long-term activation of glial cells, causing a gradual loss of neuronal function [20]. When microglia are activated, they release various harmful factors, which exert stress on neurons. Although they may not kill them, they become more susceptible to phagocytosis by activated microglia [21, 22]. A genome-wide association study found 40 risk genes associated with AD in European populations. Based on the heredity of single nucleotide polymorphisms, microglia may be the most relevant cell type affected by the disease [23].

Neuroinflammatory signals are often activated in response to pathogens or foreign substances. Because mitochondria are derived from bacteria, the immune system erroneously recognizes mitochondrial-derived DAMPs as bacteria, which triggers an innate immune mechanism [24]. When severely damaged mitochondria cannot be removed properly by mitophagy, they release their contents into the cytoplasm and extracellular environment, thereby enhancing the neuroinflammatory process. Recently, studies have demonstrated that immunity induced by mitochondrial stress is essential for effective antimicrobial defense. However, under pathological conditions, injured mitochondria may lead to abnormal activation of the innate immune system and result in autoimmune inflammation or autoimmune diseases [25]. Mitochondria contain several proinflammatory molecules, including but not limited to mtDNA [26]. These molecules can enhance the inflammatory response. Here, we describe the role of mtDNA, mitochondrial unfolded protein response (mtUPR), mitochondrial ROS (mtROS), adenosine triphosphate (ATP), transcription factor A mitochondria (TFAM), cardiolipin, cytochrome *c*, mitochondrial Ca^2+^ and iron in the neuroinflammatory response.

## mtDNA

mtDNA consists of multicopy, circular DNA of ~16.6 kb containing 37 genes [27]. mtDNA encodes protein subunits that are essential for oxidative phosphorylation (OXPHOS). Compared with nuclear DNA, double-stranded mtDNA has stronger resistance to nuclease degradation and is not easily degraded [28]. mtDNA is known to accumulate mutations with age, extensive deletions and point mutations. Damage to mtDNA is primarily caused by oxidative injury. Mitochondria produce a large amount of ROS during OXPHOS, which renders mtDNA more susceptible to oxidative damage. The rate of mutation is 10–200 times higher than that of the nuclear genome [29]. The accumulation of mtDNA mutations is associated with aging and age-related diseases [30].

mtDNA regulates energy production and cell metabolism and is a key signaling molecule that triggers inflammatory responses. mtDNA lacks protective histones and complex DNA repair mechanisms; it is therefore particularly susceptible to destructive mitochondrial factors, such as ROS. Severe mitochondrial dysfunction is characterized by the opening of mitochondrial permeability transition pores, swelling of the mitochondria and decreased mitochondrial membrane potential [31]. During stress, mtDNA is released from the mitochondria and is a significant activator of inflammation. The mechanism of mtDNA release primarily depends on the mitochondrial permeability transition pores, BAK and BAX [32]. The BAK and BAX proteins are two pro-apoptotic BCL-2 family members. Using super-resolution imaging, it was determined that the outer mitochondrial membrane is permeabilized and that BAK and BAX mediate the gradual widening of the outer mitochondrial membrane, which enables the inner mitochondrial membrane to squeeze into the cytoplasm. When BAK and BAX are activated, they oligomerize in the outer mitochondrial membrane, resulting in the appearance of large BAK/BAX pores. These macropores allow the inner mitochondrial membrane to protrude into the cytoplasm, carrying mitochondrial matrix components, including the mitochondrial genome [33].

**Fig. 1:**
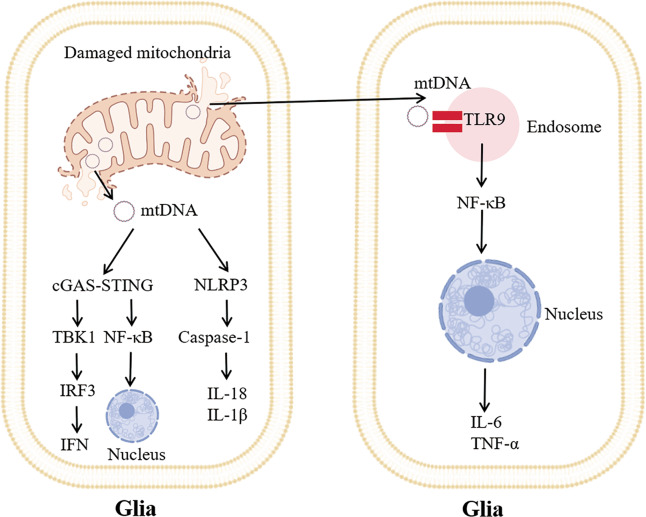
mtDNA acts as a DAMP to activate neuroinflammation. Mitochondrial dysfunction causes mtDNA to flow into the cytoplasm and out of glia (the left cell). mtDNA in the cytoplasm is sensed by the cGAS-STING signaling pathway, which induces TBK1-IRF3-IFN signaling and promotes the expression of inflammatory factors. mtDNA in the cytoplasm can also activate the NLRP3 inflammasome and promote the expression of IL-18 and IL-1β by activating caspase-1. Extracellular mtDNA activates TLR9 on the endosomes of other glia (the right cell) to initiate nuclear transcription factor NF-κB signaling.

Defective mitochondria and mtDNA are degraded by mitophagy, but after extensive mitochondrial damage, mtDNA can escape this pathway. mtDNA acts as a DAMP to regulate the inflammatory response by triggering interferon (IFN) genes, TLR9 and the NLRP3 inflammasome (Fig. 1) [34]. Mitochondrial stress and injury result in mtDNA release into the cytoplasm, activation of the DNA-induced cyclic GMP-AMP synthase (cGAS)-cyclic GMP-AMP (cGAMP)-stimulator of interferon genes (STING) pathway and promotion of the inflammatory response [35]. mtDNA derived from damaged mitochondria acts as a ligand for STING through cGAS [15]. cGAS is a double-stranded DNA sensor that detects DNA in the cytoplasm and generates cGAMP [36]. cGAMP subsequently binds directly to STING and further recruits and activates tank-binding kinase 1 (TBK1) through its C-terminal PLPLRT/SD sequence. TBK1 phosphorylates interferon regulatory factor 3 (IRF3) to trigger dimer formation and translocation to the nucleus, where it induces the expression of various IFN-stimulated genes. Moreover, TBK1 also activates the NF-κB signaling pathway through phosphorylation, thus increasing the expression of IL-6 and TNF-α.

mtDNA contains an unmethylated CpG-DNA sequence. These sequences are TLR9 ligands and can mediate inflammation through the NF-κB pathway. TLR9 is mainly located on the surface of the endosome. TLR9 recognizes the hypomethylated CpG motif in prokaryotes and senses nucleic acids. A downstream signaling cascade is then triggered through MyD88, which activates MAPK and the nuclear transcription factor NF-κB to promote inflammation.

mtDNA activates the NLRP3 inflammasome, which triggers the production of IL-18 and IL-1β [15]. Studies have demonstrated that long-term treatment with low doses of ethidium bromide damages mtDNA instead of nDNA, indicating that mtDNA is also essential for NLRP3 activation [37]. Activation of NLRP3 enhances lysis, activation of pro-caspase-1 and the production of IL-18 and IL-1β, thereby inducing inflammation [38].

Additional evidence supports a complex link between mitochondrial dysfunction and neuroinflammation, which are the leading causes of neurodegenerative diseases. Inflammatory mediators produced by activated microglia and infiltrating immune cells trigger an intracellular signaling cascade. Because of the continuous production of toxic mediators such as ROS and cytokines, chronic microglial activation eventually results in neuronal damage. Given the ability of these molecules to inhibit and destroy mitochondria, it is conceivable that neuroinflammation affects mitochondria and alters their function [39].

## Mitochondrial unfolded protein response

The mitochondrial genome encodes only 1% of the mitochondrial protein, and the rest is encoded by the nucleus [40]. mtDNA mainly encodes proteins involved in OXPHOS. The base deletions of mtDNA accumulate with aging, leading to the accumulation of misfolded proteins. The accumulation of mtDNA mutations results in a mixture of wild-type and mutant mitochondrial genomes. This phenomenon is also called mtDNA heterogeneity [41]. ROS and other harmful factors produced by OXPHOS can also damage the mitochondrial genome. Therefore, there must be a mechanism to protect the mitochondrial proteome. Mitochondria have specialized molecular chaperones to promote the correct folding of proteins and proteases to degrade misfolded proteins. When the accumulation of unfolded proteins exceeds the mitochondrial load, mitochondria initiate the mtUPR [42]. Stressed mitochondria send signals to the nucleus and transcriptionally regulate genes that help restore mitochondrial activity. ATFS-1 is a transcription factor that is mainly responsible for regulating the communication between mitochondria and the nucleus during the UPR [43]. ATFS-1 has a mitochondrial localization sequence and a nuclear localization sequence. Under physiological conditions, ATFS-1 is transported to the mitochondria and degraded. During mitochondrial stress, the mitochondrial input of ATFS-1 is impaired, allowing them to be transported to the nucleus [44]. The nuclear accumulation of ATFS-1 promotes the recovery of OXHPOS during the UPR. ATFS-1 also upregulates the expression of glycolytic pathway genes to ensure that cells have enough ATP to promote the survival of mitochondria when OXPHOS is abnormal.

ATFS-1-mediated mtUPR promotes the recovery of defective mitochondria. The initial response of the UPR helps restore cell balance, and long-term imbalance can lead to maladaptation. Recent studies have shown that the mitochondrial UPR is a double-edged sword. MtUPR activation inadvertently maintains and spreads heterogeneous mtDNA of *Caenorhabditis elegans* [45]. By inhibiting ATFS-1, the UPR is attenuated, and the number of copies of base-deleted mtDNA (ΔmtDNA) is reduced. The increased synthesis of ATFS-1 promotes the accumulation of ΔmtDNA, which further reduces the efficiency of OXPHOS [46]. The activation of ATFS-1 creates an environment conducive to ΔmtDNA. The mtUPR attempts to restore OXPHOS activity, but will instead inadvertently spread harmful ΔmtDNA. These studies revealed the unexpected and harmful consequences of mtUPR by stimulating mitochondrial biogenesis and mitochondrial dynamics to restore the activity of OXPHOS. In addition to inducing mitochondrial protection genes, ATFS-1 also upregulates the expression of innate immunity genes during mitochondrial stress [47]. ATFS-1 upregulates the p38-mediated innate immune pathway to prevent a variety of exogenous stresses [48]. A newly published document shows that mild damage to mitochondrial function can extend the lifespan of model organisms. Disruption of mitochondrial electron transport chain (ETC) subunits leads to the upregulation of mtUPR-driven and p38-mediated immune signaling pathways. These two approaches work together to promote cell resistance to pathogens [49]. When the UPR is activated, NF-κB is also upregulated [50]. Disease-specific protein misfolding is a hallmark of most neurodegenerative diseases, and the UPR may play a key role in this process. However, there are few studies on the role of the mtUPR in neuroinflammation. Therefore, in-depth exploration of the connection between the mtUPR and neuroinflammation may provide a new target for the treatment of neurodegenerative diseases.

## Mitochondrial reactive oxygen species

Mitochondrial ETC and mitochondrial NADPH oxidase (NOX) are the main producers of ROS in mitochondria. In the process of electron transfer, mitochondria continuously metabolize oxygen and produce ROS. Superoxide anion (·O_2_^−^) is the main ROS. NOX uses NADPH as a substrate to generate ROS. Since NOX is also abundant in mitochondria, the accumulation of mtROS comes from the combined action of ETC and NOX. NADPH has a dual role in cells. On the one hand, it is necessary for glutathione to maintain its reduced form, which helps cells resist oxidative stress damage. On the other hand, NADPH can be used by NOX to generate ROS. In previous studies in our laboratory, it was found that exogenous supplementation with NADPH can resist mitochondrial dysfunction in ischemia-reperfusion injury and excitotoxicity [51]. However, NADPH has the disadvantage of a narrow therapeutic window. Therefore, we used a mitochondrial-targeted NOX inhibitor and found that the combined use of NADPH and a mitochondrial-targeted NOX inhibitor can better reduce the production of mitochondrial superoxide and protect mitochondrial function [52]. Our research indicates that the use of mitochondrial-targeted antioxidants to promote the maintenance of mitochondrial function may be considered a promising aspect of treating neurodegenerative diseases.

Cells have a series of antioxidants to scavenge free radicals, including enzymatic and nonenzymatic mechanisms. Mitochondrial damage, including oxidative damage, can lead to an imbalance between ROS production and ROS removal, ultimately resulting in net ROS production. MtROS destroy proteins and lipids, and when they accumulate, they even destroy mitochondria [53]. Studies have revealed that mtROS are related to the causes of aging and other chronic diseases [54]. In the early stages of multiple sclerosis, mtROS stimulate the NLRP3 inflammasome and form local inflammatory lesions [55].

**Fig. 2:**
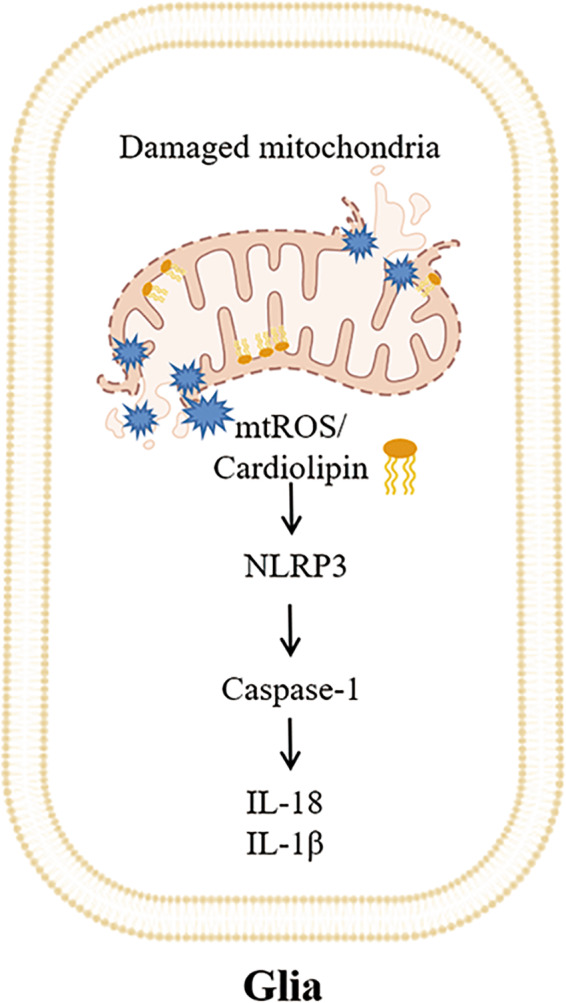
mtROS and cardiolipin act as DAMPs to activate neuroinflammation. When damaged, mitochondrial dysfunction of glia leads to excessive production of mtROS and the release of cardiolipin located on the inner mitochondrial membrane into the cytoplasm. mtROS or cardiolipin activates the NLRP3 inflammasome to initiate a caspase-1-dependent neuroinflammatory response and release IL-18 and IL-1β.

In recent years, extensive evidence has confirmed the role of mtROS in NLRP3 activation (Fig. 2). A specific mtROS scavenger, mito-Tempo, reduces the release of ROS, inhibits the activation of the NLRP3 inflammasome, and reduces the upregulation of IL-1β and IL-18 induced by ethanol or LPS [56]. The activation of NLRP3/caspase-1 induced by ethanol is mediated by mtROS because when the specific mtROS scavenger mito-Tempo is used, the release of mtROS is eliminated [56]. The common feature of NLRP3 inflammasome activators is that they are accompanied by the production of mtROS, which indirectly indicates that ROS are an upstream mediator required for the activation of NLRP3 inflammasomes [57].

Under normal conditions, the NLRP3 inflammasome is located in the endoplasmic reticulum. When inflammation is activated, NLRP3 redistributes near the nucleus, connects with the mitochondria, and starts to produce inflammasomes when ROS are produced. This suggests that the NLRP3 inflammasome senses mitochondrial dysfunction and may explain the frequent association between mitochondrial damage and inflammatory diseases [58]. Nod-like receptors recognize mtROS, which are actively induced to form the NLRP3 inflammasome. NLRP3 activates caspase-1, leading to the proteolysis and maturation of the proinflammatory cytokine IL-1β.

In astrocytes, inflammatory stimuli also induce mitochondrial fragmentation, which impairs mitochondrial energy production and further increases the production of mtROS [59]. The increase in mtROS production causes mtDNA damage. Oxidative DNA damage was detected in LPS-induced neuroinflammation; however, there was no nuclear DNA damage [60]. Studies have also demonstrated that an increase in TNF-α levels, either endogenous or exogenous, causes significant changes to mitochondria [61].

## ATP

In the tricarboxylic acid cycle, the electrons provided by the coenzymes NADH and FADH_2_ are transferred to the ETC. Electron transfer establishes an electrochemical gradient that produces ATP. ATP is the most direct energy source in organisms. Neurons depend on the normal function of mitochondria, because mitochondrial ATP is critical for synaptic assembly and action potential transmission. ATP is stored in the presynaptic vesicles and granules of healthy neurons and glial cells [62].

ATP and adenosine are important endogenous signaling molecules in immune and inflammatory responses [63]. Mixed cultures of glial cells exposed to ATP showed increased expression of IL-6 [26]. Extracellular ATP is an activator of microglia and is involved in the pathogenesis of AD [62]. Extracellular ATP stimulates the purinergic receptors (PRs) of microglia and mediates the release of proinflammatory cytokines at the injury site, leading to behavioral disorders and neurodegeneration. Acute harmful events or chronic neurodegenerative diseases can cause large amounts of ATP to be released through the leaking plasma membrane of nerve tissue. There is convincing evidence that ATP is released from the Panx1 channel into the external environment [64].

**Fig. 3:**
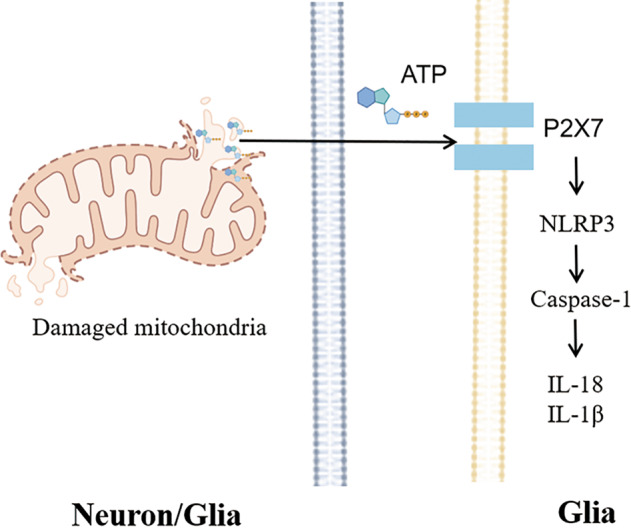
ATP acts as a DAMP to activate neuroinflammation. Under stress conditions, ATP from neurons or glia (the left cell) is released outside the cell. ATP activates the caspase-1 signaling cascade mediated by NLRP3 through the P2X7 receptor on the membrane surface of glia (the right cell), leading to neuroinflammation.

PR includes two types, ionotropic purinergic P2X and metabotropic purinergic P2Y receptors [65]. P2X7 is an ion receptor mainly located on microglia. The purinergic P2X7 receptor is an ATP-gated ion channel whose activation is associated with inflammation. P2X7 receptors are preferentially localized in microglia, and the density of astrocytes and oligodendrocytes is relatively low. The P2X7 receptor allows nonselective Na^+^/Ca^2+/^K^+^ transmembrane flux and allows the slow penetration of larger organic molecules [66]. ATP induces the activation of NLRP3 inflammasomes through the participation of P2X7 receptors (Fig. 3). P2X7 is activated, allowing Na^+^/Ca^2+^ to flow in and K^+^ to flow out. The decrease in K^+^ ions in the cell further activates NLRP3. Studies have also shown that P2X7R agonists stimulate the morphological maturation of dendritic cells and promote the expression of NF-κB, as well as the release of IFN-γ and IL-12. P2X7R inhibitors have the opposite effect. Extracellular ATP promotes the maturation of dendritic cells and the release of inflammatory factors through the combination of P2X7R and NF-κB. Coimmunoprecipitation experiments verified the interaction of P2X7R and NF-κB [67].

Compared with other cells, neurons mainly rely on mitochondrial OXPHOS to meet their energy requirements. The brain accounts for only 2% of body weight, but it consumes 20% of the total ATP [68]. Therefore, mitochondria must provide adequate amounts of ATP for neurons to function normally, and ATP is primarily derived from mitochondrial OXPHOS.

Classically activated M1-type microglia enhance aerobic glycolysis and the pentose phosphate pathway, which results in a decrease in the mitochondrial tricarboxylic acid cycle. Studies have demonstrated that LPS triggers TLRs, resulting in the phosphorylation of cytochrome *c* oxidase and other mitochondrial targets. This reduces the mitochondrial membrane potential, damages mitochondrial OXPHOS and ultimately causes energy depletion [69]. Activation of TLRs also results in the opening of mitochondrial permeability transition pores, which decreases mitochondrial oxygen consumption [70].

Inflammatory factors, especially TNF-α, inhibit mitochondrial OXPHOS, convert ATP production from OXPHOS into glycolysis, and stimulate mtROS production. This results in changes to mitochondrial energy metabolism and mitochondrial membrane permeability, leading to cell death. TNF-α and IL-1β downregulate pyruvate dehydrogenase activity and inhibit mitochondrial respiration [71]. TNF-α promotes tyrosine phosphorylation of cytochrome *c* oxidase subunit I and inhibits the activity of cytochrome *c* oxidase. Inhibition of cytochrome *c* oxidase results in decreased cellular energy. The inability to support mitochondrial function eventually results in cell death [71].

## TFAM

TFAM is a nuclear coding protein that has an important role in mtDNA metabolism. TFAM, a mtDNA-binding protein, is essential for maintaining mitochondrial genome stability. TFAM regulates packaging, stability, and replication to determine mitochondrial genome abundance [72]. TFAM is a member of the HMGB family of highly conserved and ubiquitous DNA binding proteins [73]. In addition to its intracellular functions, HMGB1 can mediate the activation of extracellular innate immune responses, including the release of chemokines and cytokines. TFAM and HMGB1 share 76% sequence homology, which implicates TFAM in the activation of glial cells [74].

**Fig. 4:**
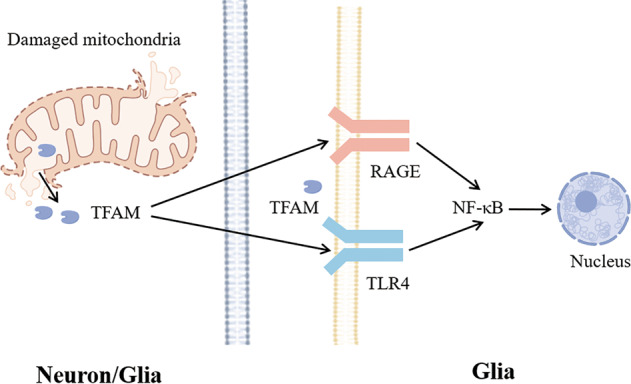
TFAM acts as a DAMP to activate neuroinflammation. Under stress conditions, TFAM from neurons or glia (the left cell) is released outside the cell. TFAM activates RAGE or TLR on the membrane surface of glia (the right cell) and initiates the NF-κB-mediated nuclear signaling cascade.

Under physiological conditions, TFAM is located in the inner mitochondrial membrane; however, when mitochondria are damaged, TFAM is also released extracellularly. One study demonstrated that TFAM induces inflammatory and cytotoxic reactions of glial cells in vivo and in vitro. Injection of TFAM into the cisterna magna of SD rats upregulated the levels of the proinflammatory factors monocyte chemotactic factor 1 (MCP-1), NF-κB, IL-6 and TNF-α in the hippocampus [75]. TFAM is an endogenous danger signal that promotes the release of TNF-α from reactive dendritic cells through TLR4 and advanced glycation end-products (RAGE) (Fig. 4). After exposure to TFAM in vitro, these same inflammatory mediators are upregulated in rat microglia. TFAM acts as a DAMP for proinflammatory microglial activation by interacting with the receptor of RAGE and macrophage antigen 1 (Mac-1). By specifically blocking RAGE and Mac-1 receptors, the inhibition of MCP-1 induced by TFAM is inhibited by monocytes. These cells can mimic human microglia. These data support the hypothesis that TFAM interacts with RAGE and Mac-1 to activate proinflammatory microglia [75]. Thus, the RAGE axis has a role in neuroinflammation and neurodegeneration. RAGE activation triggers an increase in proinflammatory cytokine production and exerts harmful effects by binding to proinflammatory ligands and through subsequent activation of downstream regulatory pathways [76].

## Cardiolipin

Cardiolipin is a component of the mitochondrial inner membrane. It has a unique dimer structure consisting of two phosphate residues, which are connected by glycerol bridges and exhibit unique physical and chemical properties [77]. Cardiolipin interacts with several inner mitochondrial membrane proteins and is required for the optimal activity of proteins, which include ETC proteins and enzyme complexes that produce ATP. Cardiolipin also plays a role in mitochondrial membrane morphology, stability and kinetics, mitochondrial biogenesis and steps associated with mitochondria and apoptosis.

Cardiolipin primarily exists in the mitochondria; however, after mitochondrial damage and depolarization, most cardiolipin is exposed to the mitochondrial surface. Depending on the amount, it can exert pro-mitochondrial or pro-apoptotic signals to cause mitochondrial damage. Cardiolipin is involved in the pathogenesis of neurodegenerative diseases. Extracellular cardiolipin can regulate microglial function by increasing microglial phagocytosis, regulating the secretion of inflammatory mediators and promoting neuroprotective effects mediated by microglia [78]. Recent studies have shown that extracellular cardiolipin regulates microglial phagocytosis and cytokine secretion in a TLR4-dependent manner. Exposure of human microglia-like cells to extracellular cardiolipin induces the secretion of MCP-1, IFN-γ and NO [79]. Studies have shown that NLRP3 is associated with mitochondria when activated. In an isotope labeling study, following NLRP3 agonist treatment, cardiolipin was directly bound to NLRP3 and responsible for the activation of NLRP3 [57]. Static NLRP3 is located in the endoplasmic reticulum structure, and when the inflammasome is activated, NLRP3 and its linker ASC redistribute to the perinuclear space, where they colocalize with the endoplasmic reticulum and mitochondrial organelle clusters [80]. Cardiolipin binds to mitochondria-located NLRP3 and activates the NLRP3 inflammasome, resulting in a neuroinflammatory response (Fig. 2) [81].

Mitochondrial dynamics include mitochondrial fission/fusion, mitochondrial transport, and mitophagy. Mitochondrial dynamics play an important role in eliminating damaged mitochondria and maintaining mitochondrial balance. The balance of mitochondrial fission and fusion maintains the homeostasis of the mitochondrial structure and the distribution of mitochondrial components. The homeostasis of mitochondrial fission and fusion in astrocytes is altered during neuroinflammation. Proinflammatory stimulation causes local changes in mitochondrial dynamics and is more likely to induce mitochondrial fission than fusion. This effect is triggered by increased phosphorylation of DRP1 (Ser616), leading to excessive mitochondrial fission and mitochondrial fragmentation [82]. Moreover, the increase in mtROS production and autophagy significantly decreases mitochondrial respiratory capacity [59].

Autopsy reports of patients with AD have confirmed a decrease in Mfn1/Mfn2 and OPA1 levels and increased FIS1 and DRP1 levels. This suggests that an imbalance of mitochondrial fission/fusion contributes to AD. Andre’s study demonstrated that increased expression of the proinflammatory factor IL-1β in patients with AD mediates changes in mitochondrial fission/fusion protein levels. After treatment with the mitochondrial fission inhibitor, Mdivi-1, IL-1β expression in the hippocampus was downregulated. This indicates that negative feedback regulation exists between mitochondrial dynamics and IL-1β-mediated inflammation. Mitochondrial dynamics and neuroinflammation are bidirectionally regulated in their involvement in synaptic loss, resulting in memory decline [83].

## Cytochrome *c*

**Fig. 5:**
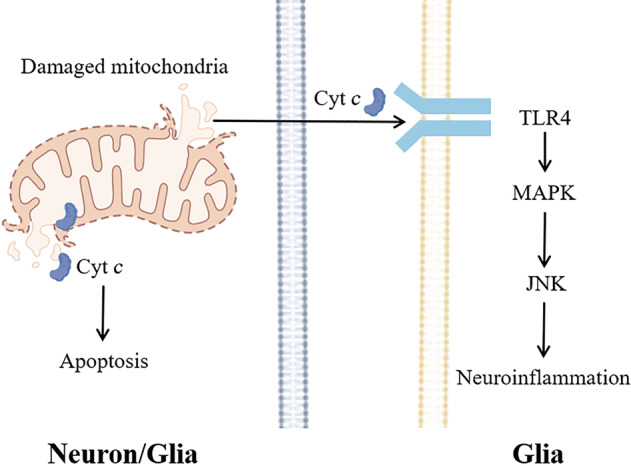
Cytochrome *c* acts as a DAMP to activate neuroinflammation. Under stress conditions, the mitochondrial membrane of neurons or glia (the left cell) ruptures and cytochrome *c* is released outside the mitochondria. Cytochrome *c* in the cytoplasm activates the apoptosis signaling pathway. Extracellular cytochrome *c* activates the MAPK-JNK signaling cascade through TLR4 on the membrane surface of glia (the right cell), leading to neuroinflammation.

Cytochrome *c* is a mitochondrial respiratory chain protein that is located between the inner and outer mitochondrial membranes. As the electron carrier between ETC complexes III and IV, cytochrome *c* acts as an ETC carrier and ROS scavenger [84]. Earlier, we focused on the role of cytochrome *c* in apoptosis. When cytochrome *c* is released into the cytoplasm, it initiates apoptosis. Recent studies have demonstrated that cytochrome *c* is also released into the extracellular space by healthy or damaged cells. An increase in the concentration of cytochrome *c* was observed in cerebrospinal fluid, indicating that cytochrome *c* may also be released into the extracellular space by damaged cells [85]. Cytochrome *c* released outside of the mitochondria interacts with microglial TLR4 receptors and the C-Jun N-terminal kinase (JNK) signaling pathway to regulate the function of immune cells in the brain (Fig. 5) [86].

Damaged glial cells are a potential source of extracellular cytochrome *c* in the CNS. Cytochrome *c* can be released from damaged glial cells into the extracellular space, thereby activating the immune activity of astrocytes in a TLR4-dependent manner. Cytochrome *c* induces human astrocytes to secrete IL-1β and IL-8, and anti-TLR4 antibodies can block this change. This confirmed an interaction between cytochrome *c* and astrocyte TLR4 [87]. Adding cytochrome *c* to cultured primary human astrocytes increases the secretion of IL-1β and granulocyte-macrophage colony-stimulating factor [87]. The core event of many DAMP-mediated immune responses is the activation of the MAPK signaling cascade. JNK is a mature downstream signaling molecule that is associated with TLR4 activation. Selective inhibition of JNK attenuates the production of nitrite in BV-2 cells. This indicates that the function of cytochrome *c* as a DAMP may depend on the activation of the JNK pathway [86]. Activation of astrocytes by cytochrome *c* may cause neuroinflammation and neuronal death in neurodegenerative diseases. Therefore, astrocyte TLR4 may be a potential therapeutic target for degenerative diseases.

## Ca^2+^ and iron

In addition to the abovementioned common mitochondrial-derived DAMPs, recent studies have shown that cellular calcium and iron ions are also closely related to inflammatory pathways. Mitochondria are responsible for buffering the level of Ca^2+^ in the cytoplasm [88]. Under physiological conditions, the inner mitochondrial membrane is almost impermeable to metabolites and ions. However, under conditions of high Ca^2+^, the nonspecific pores of the mitochondria can be opened, which destroys the permeability barrier of the inner mitochondrial membrane. Ca^2+^ overload eventually results in mitochondrial dysfunction [89]. Studies have shown that Ca^2+^ signaling and neuroinflammatory signaling mechanisms exhibit extensive crosstalk [90, 91]. The production of cytokines in glial cells is strongly dependent on the Ca^2+^-dependent protein phosphatase calcineurin [92]. Calcineurin can upregulate a variety of cytokines and proinflammatory factors in immune cells [93]. Moreover, proinflammatory factors can increase the activity of L-type voltage-sensitive Ca^2+^ channels in neurons, leading to a cellular Ca^2+^ imbalance.

Because ferric iron is mostly insoluble, bioavailable iron primarily exists as ferrous iron. Mitochondria utilize a significant amount of iron. Extracellular iron is taken up by cells and transported to the mitochondria, where it is used to synthesize cofactors that are essential for the function of enzymes involved in redox reactions, DNA synthesis and repair and various other processes. The biosynthesis of heme occurs only in the mitochondria. Studies have found that ferritin levels are elevated in PD models, which suggests that changes in iron levels may play a role in the pathogenesis of PD [94]. Ferroptosis causes the release of mitochondrial DAMPs and neuroinflammation. Iron can induce the proinflammatory M1 phenotype of microglia, which is inhibited by NAC. Iron overload can transform macrophages into a proinflammatory phenotype through the ROS/acetyl-p53 pathway [95].

## Conclusion

**Fig. 6:**
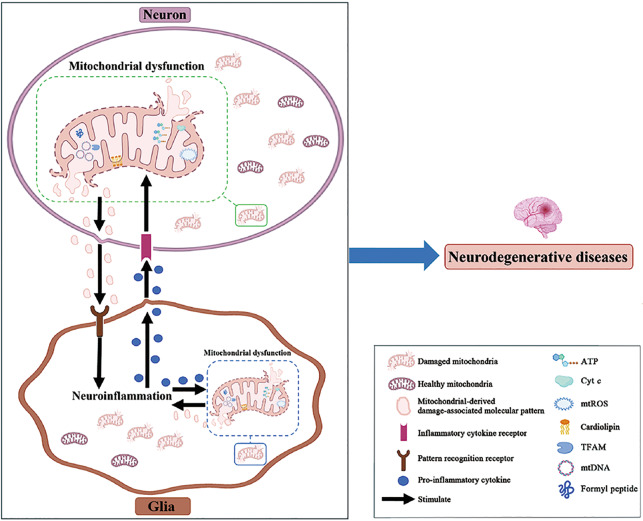
Cross-talk between mitochondrial dysfunction and neuroinflammation in neurodegenerative diseases. After exposure to external stimuli, the function of mitochondria in neurons or glia is impaired, which results in a decrease in mitochondrial membrane potential. Some molecules that originally existed inside the mitochondria, such as mtDNA and cardiolipin, were released into the cytoplasm or even outside the cell. These released molecules, also called damage-associated molecular patterns, interact with pattern recognition receptors on glia to activate inflammatory pathways. Activated glia release proinflammatory factors, which aggravate mitochondrial damage, forming a vicious cycle of mitochondrial dysfunction and neuroinflammation. The interaction between mitochondrial dysfunction and neuroinflammation ultimately results in neurodegenerative diseases. ATP adenosine triphosphate, Cyt *c* cytochrome *c*, mtDNA mitochondrial DNA, mtROS mitochondrial reactive oxygen species, TFAM mitochondrial transcription factor A.

Mitochondrial dysfunction and neuroinflammation are pathological features of neurodegenerative diseases. However, they are not independent in diseases but have complex connections. Most studies suggest that mitochondrial dysfunction occurs before neuroinflammation and plays a role in promoting neuroinflammation. In this review, we focus on the impact of mitochondrial dysfunction on neuroinflammation (Fig. 6). We are more inclined to think that mitochondrial dysfunction occurs before neuroinflammation and can cause neuroinflammation in neurodegenerative diseases. Gila can easily recognize mitochondrial-derived molecules such as mtDNA, TFAM and cytochrome *c* as harmful substances, thereby activating neuroinflammation. However, whether there is a causal relationship and a cascade relationship between mitochondrial dysfunction and neuroinflammation is still very controversial. At the same time, it is still unclear whether blocking the interactive signaling pathway between mitochondrial dysfunction and neuroinflammation can become a therapeutic target. Therefore, we need to explore the molecular mechanism of the interaction between mitochondrial dysfunction and neuroinflammation to determine the relationship between them. In the future, we can start with the key signaling molecules related to mitochondrial dysfunction and neuroinflammation, and find more effective targets for the treatment of neurodegenerative diseases.
